# Detecting early signs of heat and drought stress in *Phoenix dactylifera* (date palm)

**DOI:** 10.1371/journal.pone.0177883

**Published:** 2017-06-01

**Authors:** Omid Safronov, Jürgen Kreuzwieser, Georg Haberer, Mohamed S. Alyousif, Waltraud Schulze, Naif Al-Harbi, Leila Arab, Peter Ache, Thomas Stempfl, Joerg Kruse, Klaus X. Mayer, Rainer Hedrich, Heinz Rennenberg, Jarkko Salojärvi, Jaakko Kangasjärvi

**Affiliations:** 1 Department of Biosciences, University of Helsinki, Helsinki, Finland; 2 Institute of Forest Sciences, University of Freiburg, Freiburg, Germany; 3 Helmholtz Zentrum München, German Research Center for Environmental Health (GmbH), Plant Genome and Systems Biology, Neuherberg, Germany; 4 College of Sciences, King Saud University, Riyadh, Saudi Arabia; 5 Institute for Physiology and Biotechnology of Plants, Plant Systems Biology, University of Hohenheim, Stuttgart, Germany; 6 Institute for Molecular Plant Physiology and Biophysics, University of Würzburg, Würzburg, Germany; 7 Center of Excellence for Fluorescent Bioanalytics (KFB), University of Regensburg, Regensburg, Germany; USDA Agricultural Research Service, UNITED STATES

## Abstract

Plants adapt to the environment by either long-term genome evolution or by acclimatization processes where the cellular processes and metabolism of the plant are adjusted within the existing potential in the genome. Here we studied the adaptation strategies in date palm, *Phoenix dactylifera*, under mild heat, drought and combined heat and drought by transcriptomic and metabolomic profiling. In transcriptomics data, combined heat and drought resembled heat response, whereas in metabolomics data it was more similar to drought. In both conditions, soluble carbohydrates, such as fucose, and glucose derivatives, were increased, suggesting a switch to carbohydrate metabolism and cell wall biogenesis. This result is consistent with the evidence from transcriptomics and *cis*-motif analysis. In addition, transcriptomics data showed transcriptional activation of genes related to reactive oxygen species in all three conditions (drought, heat, and combined heat and drought), suggesting increased activity of enzymatic antioxidant systems in cytosol, chloroplast and peroxisome. Finally, the genes that were differentially expressed in heat and combined heat and drought stresses were significantly enriched for circadian and diurnal rhythm motifs, suggesting new stress avoidance strategies.

## Introduction

As a result of their sessile lifestyle, plants have developed multiple strategies for coping with environmental challenges, such as biotic (for example infection or herbivore attack) or abiotic (such as heat, drought, or high light) stresses. In nature, plants experience several stresses simultaneously, resulting in limited crop yields and growth. The impact of different stress types on plants has been under extensive research, but studies looking at their combinations are still relatively few in comparison. It has turned out that combined stresses invoke responses which can be radically different from the application of individual stresses alone [1], but also that the stress types have certain shared components [2]. Response to combined stresses appears to be species specific, and in general the response is towards the more damaging stress condition [2]. Additionally, there are differences between individuals, suggesting potential for breeding [3].

Here we study the effect of heat, drought and combined heat and drought stress on date palm, *Phoenix dactylifera*. In natural conditions, heat and drought often occur at the same time, with heat enhancing the severity of drought stress. Heat stress results in the expression of heat shock proteins through a signaling cascade that is not yet fully understood, but which involves calcium signaling induced by extracellular Ca^2+^ influx, followed by H_2_O_2_ increase within approximately ten minutes after application of stress, and nitrogen oxide (NO) signaling roughly 30 min after stress [4]. An alternative hypothesis based on animal studies suggests that the accumulation of misfolded proteins in the cell membrane itself directly triggers the expression of heat shock proteins [5]. In addition to heat shock proteins, the composition of cell membrane has been shown to undergo changes in lipid composition under heat stress [6]. Furthermore, abscisic acid (ABA) is required for acquired thermotolerance.

Plants deal with different stresses either by avoiding the stressor (stress avoidance), or by gaining the abilities to maintain plant function in presence of the stressor (stress tolerance). Many plants are using both stress avoidance and tolerance strategies to cope with environmental challenges. For example drought stress usually results in several phenotypic alterations [7], which can be viewed as stress avoidance strategies. At the molecular level, drought tolerance is initiated by the production of ABA. This triggers a signaling pathway that leads into production of reactive oxygen species (ROS), an increase in cytosolic Calcium, followed by activation of ion channels to induce stomatal closure. In nucleus, various genes are expressed, including ABA biosynthesis genes, late embryogenesis abundant (LEA) genes, as well as several transcription factors, including DREB/CBF (drought-responsive *cis*-element binding protein/C-repeat-binding factor) [8].

Date palm (*Phoenix dactylifera*) is a perennial, dioecious plant of the Arecaceae family. It is among the first crops domesticated by early human civilization, with a cultivation history of over 6000 years [9]. It is distributed in arid and semi-arid regions of north Africa and the middle east, and within the last centuries it has been introduced to southeast Asia, southern Africa, Australia, south America, Mexico and the United States. It is an important agricultural crop and an important a dietary ingredient in many countries worldwide. Date palm is exceptional in the sense that it can withstand extreme temperatures (ranging from 56–60°C to few degrees below zero) and harsh climatic conditions. Understanding the strategies adapted by an extremely heat tolerant species may also help in developing more heat tolerant crops.

Here we have studied the responses of date palm to heat, drought and combined heat and drought stress, which are mild for the *P*. *dactylifera* but severe to the model plant *Arabidopsis thaliana*. We set up a controlled experiment and quantified gene expression and metabolite concentrations from paired samples. Furthermore, we carried out a *cis*-motif enrichment analysis of the genes expressed in different stresses. Significantly enriched motifs and the enriched gene ontology (GO) categories associated with the stresses are discussed in more detail.

## Materials and methods

### Plant material

The plant material was collected as described previously [10,11]. *P*. *dactylifera* seedlings were purchased from "Der Palmenmann", Bottrop, Germany. Plants were repotted with a peat–sand–perlite mixture [20:30:50 (vol%)] to which 10 gram (g) of NPK fertilizer was added, and maintained under greenhouse conditions (15–25°C, 60–70% relative humidity (rH)) two months prior to the start of the experiments. Plants were irrigated every second day towards the end of the light period. Experiments were performed in growth chambers (Heraeus, Vötsch, Germany) with 16/8 h photoperiod and 20/15°C (70 ± 3% rH) or 35/15°C (60 ± 8% rH at day and 70 ± 3% at night) temperature and rH under 200–300 mmol photons m^-2^ s^-1^ at leaf level.

Experiments were carried out in two batches. In the first set, plants were irrigated every second day ("well-watered”, heat and control conditions). Plants were first acclimatized for two weeks in the chambers and then exposed to different growth temperatures (20°C for control, and 35°C for heat) for two weeks, followed by harvesting six hours after the onset of light. In the second batch, watering was stopped after the two-week acclimation period. Plants grown at 35°C were harvested 4–5 days after termination of irrigation (combined heat and drought), and plants grown at 20°C were collected after 7–8 days (drought conditions). The duration of water deprivation was 3 days longer for 20°C-grown plants because of lower relative humidity in the 35°C chamber. In experimental conditions involving drought the leaf water content (fresh weight/dry weight ratio) was monitored, showing that the treatments caused a significant decline in leaf water content, see [11] for more details. Four plants (biological replicates) from each experimental condition were harvested for total RNA and metabolite isolation. After collection, plant material was frozen in liquid N_2_ and stored at -80°C.

### Metabolomics data

Extraction and derivatization of plant samples (same plant samples which were used for RNA extraction, with four replicates in each experiment) were performed as described in [10,12]. Briefly, leaf and root material of about 50 mg fresh weight was homogenized and extracted in 700mL 100% methanol at 70°C for 15 min and centrifuged at 14,000 rpm for 5 min. The supernatants were transferred to new tubes and 1 mL double-distilled water/chloroform were added, tubes were vigorously shaken and centrifuged at 14,000 rpm for 5 min. Aliquots of 200mL of the chloroform phase were dried in a speed-vac (RVC 2–25, Christ, Osterode, Germany) and derivatized. For derivatization 50mL methyl-N-(trimethylsilyl) trifluoroacetamide (MSTFA; Sigma, Munich, Germany) with 20mL pyridine were added and samples were incubated at 37°C for 30 min. Subsequently, sample reaction solutions were transferred to glass vials suitable for the Gerstel MultiPurpose Sampler (MPS2-XL, Gerstel, Mülheim, Germany). 1mL aliquots were injected into the system and run on a capillary column (HP-5MS, length: 30 m, diameter: 0.25 mm, film thickness: 0.25mm; Agilent Technologies, Palo Alto, CA, USA) at a helium flow of 1mLmin^-1^.

Metabolite abundance was determined by GC-EIMSD (Agilent 7890A GC coupled to an Agilent 5975C MS; Agilent Technologies, Frankfurt, Germany) with the GC/MS settings described in [13]. For metabolite identification and quantification, the Golm metabolome database [14] and available authentic external standards of known concentration were used. Peak identification and deconvolution of chromatograms were performed using AMDIS 2.71 (“Automated Mass Spectral Deconvolution and Identification System” freely available from http://www.amdis.net) and the web-based platform “SpectConnect” (http://spectconnect.mit.edu/) [15].

Differential abundance of the metabolite data was analyzed using EdgeR (v 3.14.0) [16]. Data was normalized using the default Trimmed Mean of M-values (TMM). The glmLRT method was used to fit the statistical model. Benjamini-Hochberg false discovery rate correction (FDR) of p-values was used to adjust for multiple testing, and FDR ≤ 0.05 was used as a significance threshold.

### RNA sequencing and RNAseq data processing

RNA was extracted from a single pinna leaf and cDNA preparation was carried out as described in [17]. NEBNext Ultra^™^ RNA Library Prep protocol (New England Biolabs, Ipswich, MA, USA), Illumina HiSeq 1000 System User Guide (Illumina, Inc., San Diego, CA, USA) and KAPA Library Quantification Kit—Illumina/ABI Prism User Guide (Kapa Biosystems, Inc., Woburn, MA, USA) were used in library preparation and RNA sequencing. To this end, 500 ng of total RNA were used for library preparation (NEBNext Poly(A) mRNA Magnetic Isolation Module). Later on, purified RNAs were reverse transcribed using random primers to construct forward and reverse cDNA strands. Amplified cDNAs were treated for end-repair process by addition of a single ‘A’ base, and ligation of the barcode-containing adapters (NEBNext Multiplex Oligos, New England Biolabs). Finally, treated cDNAs were purified for DNA library preparation, quantified by KAPA SYBR FAST ABI Prism Library Quantification Kit. To construct the cBot (TruSeq PE Cluster Kit v3) cluster, equimolar amounts were pooled. A 2x100 bp paired-end sequencing run was performed on a HiSeq 1000 instrument, using TruSeq SBS v3 Reagents. The output (.bcl files) were converted into.fastq files with CASAVA 1.8.2 software. Library preparation and sequencing were conducted at the Genomics Core Facility “KFB—Center of Excellence for Fluorescent Bioanalytics” at University of Regensburg, Germany.

The quality of the raw reads was checked with FastQC software (www.bioinformatics.babraham.ac.uk/projects/fastqc/). This was followed by removal of adapter sequences and trimming and cropping of the reads using Trimmomatic-0.33 [18] in paired-end mode. The bases with a Phred quality score less than 20 were trimmed from the ends of the reads, and the reads shorter than 30 bases were removed from the analysis (-phred33, TRAILING:20 and MINLEN:30).

### Differential gene expression analysis

Filtered reads were mapped to the *P*. *dactylifera* gene models (with ribosomal genes removed) [19] using Kallisto V-0.43.0 (CMD:quant) [20] with 4000 bootstrap sets. The final count table for each biological replicate was obtained as the mean of the bootstrap runs. The count table was used as input to EdgeR (v 3.14.0) [16] to carry out differential gene expression analysis. Genes with no expression were removed and the filtered count table was normalized using the default Trimmed Mean of M-values (TMM). The glmLRT method was used to fit the statistical model. Benjamini-Hochberg false discovery rate correction of p-values was used to adjust for multiple testing, with FDR ≤ 0.05 as significance threshold.

### Ortholog inference and orthologs associated with ROS responses

Protein sequences of *Arabidopsis thaliana*, *Ananas comosus*, *Oryza sativa* (japonicus), *Zea mays* and *Sorghum bicolor* were downloaded from Phytozome v.11 [21] and used for orthology analysis by running Orthofinder [22] with default parameters.

ROS signature gene sets were collected from [23,24] and annotated according to gene family and the localization of the proteins in subcellular compartments in Arabidopsis. These candidate gene sets were mapped to their putative orthologs (custom bash shell script) using the orthologous gene clusters from Orthofinder.

### Gene annotation and Gene Ontology (GO) enrichment analysis

In order to annotate the genes in date palm, the predicted protein sequences [19] were mapped to *Arabidopsis thaliana* proteome using BLASTP with a default E-value threshold of 10 [25]. The Gene Ontology categories and the functional description of the best hit in Arabidopsis was chosen to represent the annotation of the respective *P*. *dactylifera* gene.

Lists of differentially expressed genes were selected by using the absolute value of log_2_ fold change (FC) cutoffs 1 and 4, with FDR adjusted p-value threshold of <0.05.

Gene ontology enrichment analysis was carried out with GOATOOLS [26] using Fisher exact test. Multiple testing correction with Benjamini-Hochberg false discovery rate adjustment was applied for the p-values, using significance threshold FDR ≤ 0.05. A second GO enrichment analysis was carried out with threshold-free gene set enrichment analysis (GSA) using R package Piano (v 1.12.1) [27] to detect coordinated changes in gene expression (significance threshold for FDR adjusted p-values ≤ 0.05, minimum gene set size 2 with no upper bound, using maxmean as gene set statistic, and using 10,000 bootstrap sets) [27]. Enriched GOs with significantly increased mean transcript levels were grouped using the treemap package in R [28] with default parameters.

### Motif enrichment

A total of 163 known motifs were collected from AGRIS [29] and PLACE [30] databases, and various other publications [31–33] for motif enrichment analysis. Motif enrichment was tested in the promoter sequences 1 kbp upstream of *P*. *dactylifera* genes. The motif search was conducted separately for the genes on positive and negative strands (custom R script). Motif counts were tabulated, and one-sided (alternative hypothesis: greater) Fisher exact test was applied to carry out enrichment analysis. The p-values were corrected using Bonferroni correction with ≤0.05 as significance threshold. In addition, GO enrichment analysis was carried out for genes with circadian and circadian-related motifs, light responsive, and sugar biosynthesis-related motifs.

## Results and discussion

### Gene expression

**Fig 1:**
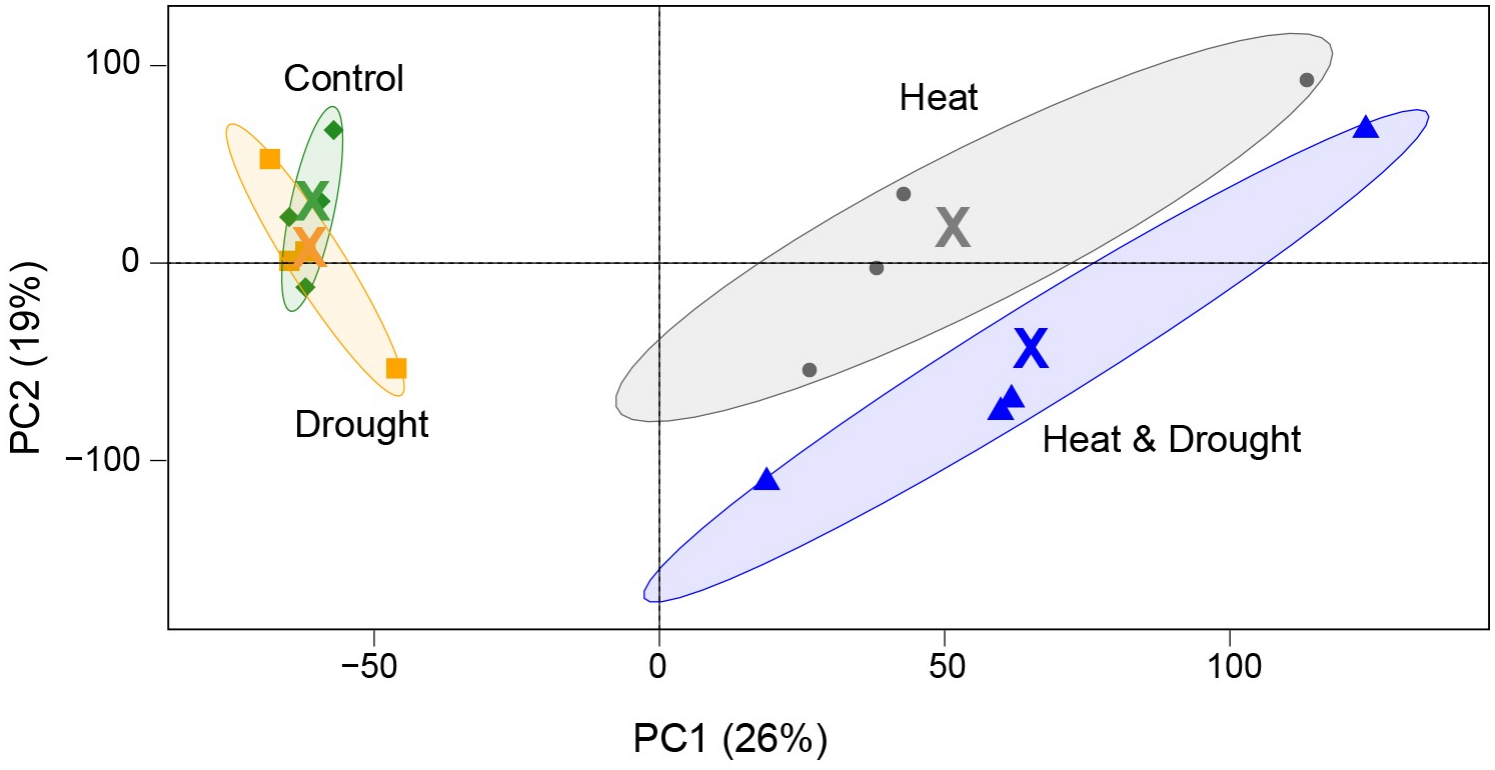
Principal component analysis of gene expression in heat, drought, combined heat and drought and control experiments. Prior to principal component analysis, genes were filtered by calculating the coefficient of determination (CoD), with the threshold of CoD > 0. The biological replicates are grouped with an ellipse, colored by experiment; green: control, dark gray: heat, yellow: drought, dark blue: combined heat and drought. The mean of four biological replicates is denoted by “X”.

Roughly 95% of all reads were retained after trimming. Information of raw and trimmed read counts per sample are collected in S1 Table. Principal component analysis (PCA) of transcriptome (Fig 1) illustrates that heat was associated with the principal component 1 (PC1), describing 26% of the total variance. In total, drought (D), heat (H) and combined heat and drought (HD) experiments resulted in 24,504 expressed genes (S5 Table).

Setting the threshold of significance to abs(log_2_FC) ≥ 1 and FDR ≤ 0.05 resulted in 68, 1240, and 3168 differentially expressed genes in drought, heat, and combined heat and drought stresses, respectively. A common factor for all treatments was the expression of genes encoding heat shock proteins (HSPs). Altogether 38 genes were differentially expressed in all three experiments (S5 Table). From this set, 19 genes were related to heat stress responses, such as heat shock proteins, chaperone proteins and heat stress transcription factors, illustrating both heat and drought induced heat-related transcriptional responses in date palm [4]. The two significantly expressed heat stress transcription factors (PDACT_KE332562.1_G003770 and PDACT_KE332562.1_G003780) were putative orthologs of Arabidopsis heat stress transcription factor A-2 (HsfA2), known to regulate the expression of a number of heat shock proteins (HSPs) in Arabidopsis and to induce the expression of L-ascorbate peroxidase 2 (APX2), Inositol-3-phosphate synthase isozyme 2 (IPS2) and galactinol synthases 1 and 2 (GolS1) [34]. Additionally, two negative regulators of cell death, orthologs of Bax inhibitor-1 family protein and Fuzzy Onions Like [35,36], were highly expressed in all three conditions suggesting suppression of cell death, as well as one positive regulator, putative ortholog of Arabidopsis BAG6 chaperone regulator [37]. In Arabidopsis, BAG6 is a positive regulator of heat shock factors and cell death. Possibly the two negative regulators are needed to prevent the cell death initiation.

Threshold-free gene set enrichment analysis [27] revealed ten enriched GOs common to heat, drought and combined heat and drought. Analysis of the enriched GO terms using Treemap [28] shows “cellular response to unfolded protein” and “induction of programmed cell death” categories to be enriched in drought and combined heat and drought (S1 and S3 Figs). A majority of the 38 genes which were differentially expressed in all conditions belonged to these two GO categories. In addition to these, pathways related to phytohormones (in H, D and HD), wax and secondary metabolites (in H and HD), fatty acid biosynthesis (in H and HD) and plant cell wall (in H, D and HD) were enriched.

### Metabolomics

**Fig 2:**
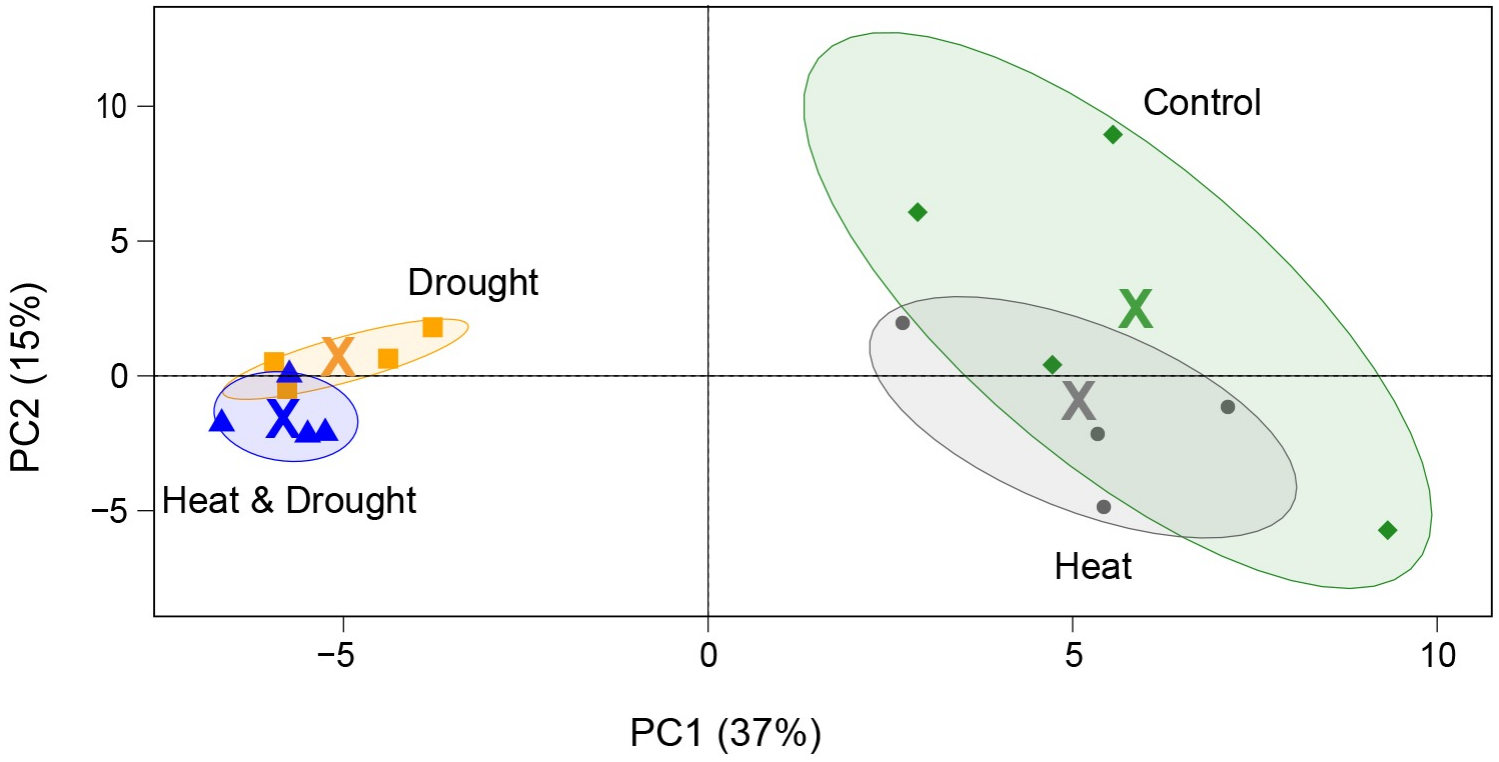
Principal component analysis of the metabolite abundances in heat, drought, combined heat and drought, and control. Biological replicates are grouped with an ellipse, coloured by experiment. Green: control, dark gray: heat, yellow: drought, dark blue: combined heat and drought. The mean of the four biological replicates is denoted by “X”.

In total, concentrations of 91 amino acids, sugars, organic acids and ten unknown metabolites were quantified (S7 Table). In contrast to transcriptomics data, the PCA plot of metabolomics data showed drought as the main descriptor of variance (37% explained by PC1, Fig 2).

**Fig 3:**
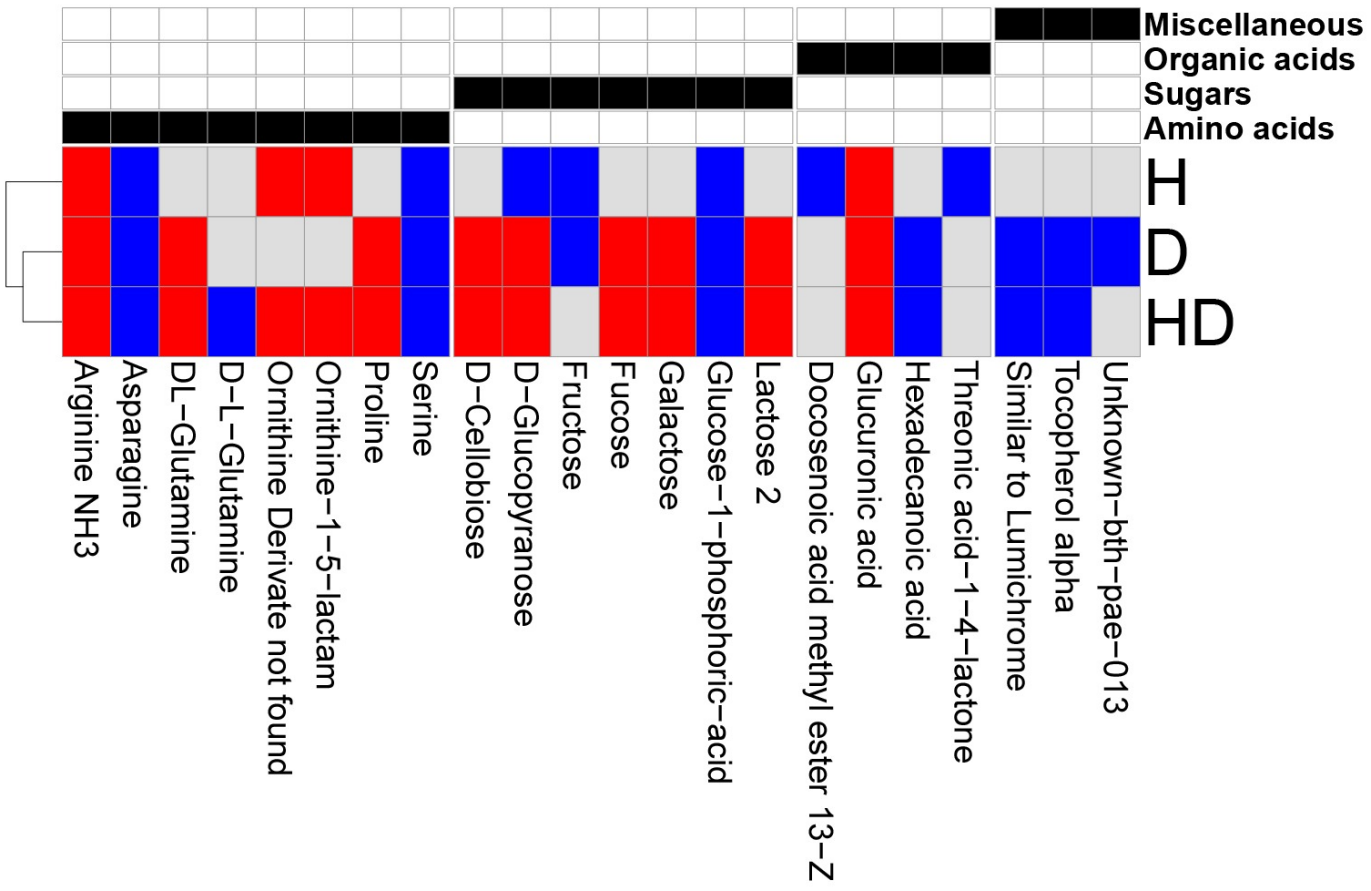
Heatmap of differentially abundant metabolites. FDR ≤ 0.05 in at least one of the treatments was used as a threshold for filtering the metabolite list for the heatmap (leaving 19 out of 91 metabolites). Columns show metabolites and rows show treatments. Each metabolite was manually annotated to be associated with biosynthesis of an organic compound, illustrated by an annotation matrix over the heatmap. Black cell shows the annotation assigned to each metabolite. Red: log_2_FC ≥ 1, blue: log_2_FC ≤ -1 with respect to control, gray: non-significant fold change (S7 Table). Abbreviations: H (heat experiment), D (drought experiment), and HD (combined heat and drought).

Overall, drought and combined heat and drought had more similar effect on metabolites. The abundance of arginine, glucuronic acid, DL-glutamine, ornithine, fucose, galactose, lactose and proline increased significantly in at least one of the conditions (Fig 3 and S7 Table). Altogether, this suggests increased activity in carbohydrate metabolism.

### Reactive oxygen species scavenging and anti-oxidative system

We next analyzed the expression of the genes related to ROS scavenging or redox-related processes, as identified in *A*. *thaliana* [23,24]. The signature sets were first mapped to sets of putative orthologs in *P*. *dactylifera* using OrthoFinder, and then these sets were tested for enrichment among the differentially expressed genes using Fisher exact test (S10 Table). The putative orthologs were subdivided by gene family and their localization in plant cell, according to information from *A*. *thaliana*.

**Fig 4:**
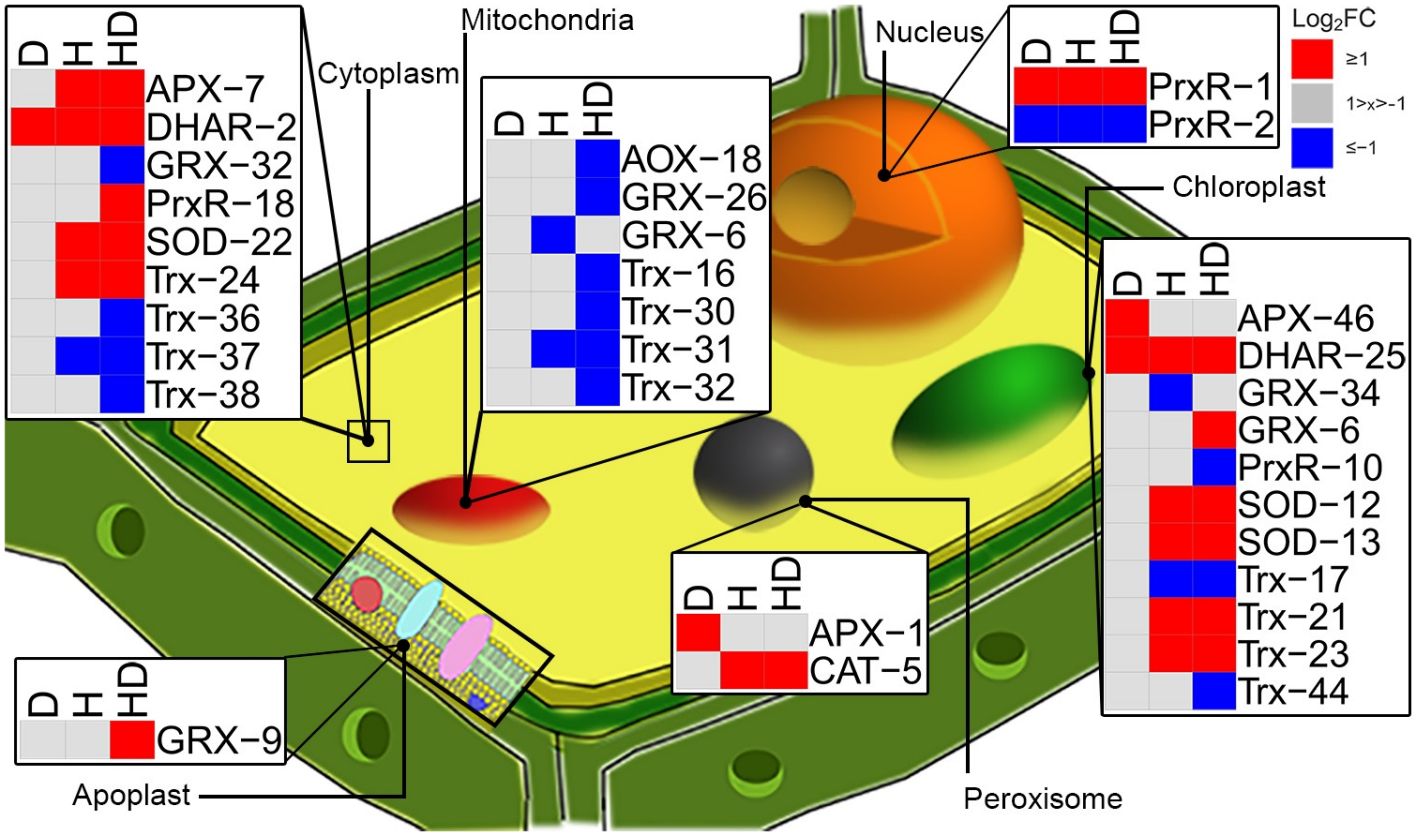
Differentially expressed genes encoding ROS and redox-related enzymes in heat, drought, or combined heat and drought treatments in *P*. *dactylifera*. Genes encoding proteins for ROS scavenging and redox-related processes were assigned to a subcellular compartment according to the protein localization in Arabidopsis, and the expression profile of the genes was used to construct the heatmaps. Genes are in rows, the number following the gene name is arbitrary and used to simplify the identification. The experiments (D: drought, H: heat and HD: combined heat and drought) are in columns. Heatmap shows log_2_FC of the expressed genes. Red: log_2_FC ≥ 1, blue: log_2_FC ≤ -1, gray: -1 < log_2_FC < 1. SOD: superoxide dismutase, AOX: alternative oxidase, APX: ascorbate peroxidase, GPX: glutathione peroxidase, CAT: catalase, GRX: glutaredoxin, Trx: thioredoxins, PrxR: peroxiredoxin and DHAR: dehydroascorbate reductase (S8 Table).

The key components of the antioxidative system known to control ROS production in plants are superoxide dismutase [SOD], glutathione peroxidase [GPX], ascorbate peroxidase [APX], catalase [CAT], Dehydroascorbate reductase [DHAR]. Additionally genes linked to redox-related processes (alternative oxidase [AOX], glutaredoxin [GRX], thioredoxin [Trx], and peroxiredoxin [PrxR]) were included. Genes encoding ROS scavenging-related proteins targeted to chloroplast and cytosol had increased expression, whereas the expression of genes encoding proteins targeted to mitochondria was decreased (Fig 4 and S8 Table).

Earlier analysis of the same experiment [10] showed that ROS homeostasis or cellular redox-state did not change in the experiment. The gene expression data however displayed changes at the transcriptional level for these processes. This suggests that ROS scavenging systems function effectively in different subcellular compartments, along with non-enzymatic ROS scavengers maintaining the ROS homeostasis. More detailed analysis on protein levels and enzyme activities will be required to analyze whether the increased transcript abundance for genes encoding antioxidative enzymes was translated into increased enzyme activities that would reflect increased ROS production by the treatments.

### Activation of pathways associated with stress tolerance

In order to detect pathways contributing to stress tolerance, gene set analysis was carried out for the transcriptomics data (S2 Table), and the results were interpreted together with the metabolite data.

#### Drought

**Fig 5:**
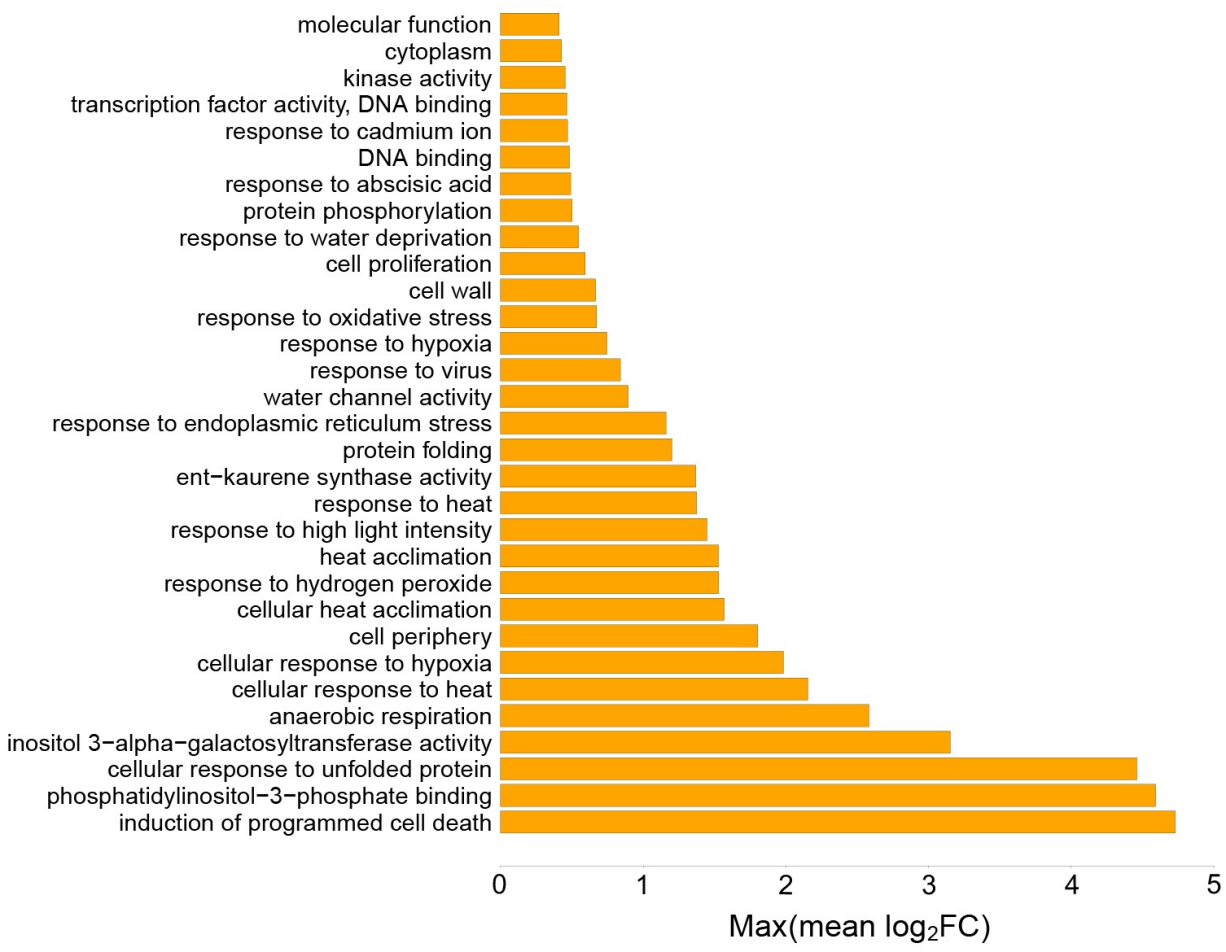
Pathways with highest differential expression levels in drought stress. Gene set analysis (GSA) identified a set of significantly enriched GOs. Y-axis shows the significantly enriched GO terms, and x-axis is the maxmean statistic of the gene set expressed in terms of log_2_FC (S2 Table).

In total, 31 pathways (out of 70 significantly enriched pathways) were identified with mean log_2_FC>0 in drought stress (Fig 5). Among them were pathways related to protein folding and unfolding, heat acclimation, water deprivation, response to ABA, hydrogen peroxide and viruses (S1 Fig).

Protein folding is vital for any organism to function and survive under stress (for example heat and drought stresses) [38]. HSPs and their chaperon activities are the main driving force behind protein folding, assembly, translocation and degradation upon normal or stressed environmental conditions [39]. “Cellular response to unfolded protein” was the major biological process enriched in response to drought (S1 Fig). It is well known that stresses (in this study H, D and HD) cause the protein folding to slow down in endoplasmic reticulum (ER), resulting in an abundance of folded, unfolded or misfolded proteins in ER [40]. The accumulation of folded, unfolded or misfolded proteins in ER may trigger a set of biological pathways such as “response to endoplasmic reticulum stress”, which was significantly enriched together with protein folding and unfolding processes in all three treatments.

#### Heat

**Fig 6:**
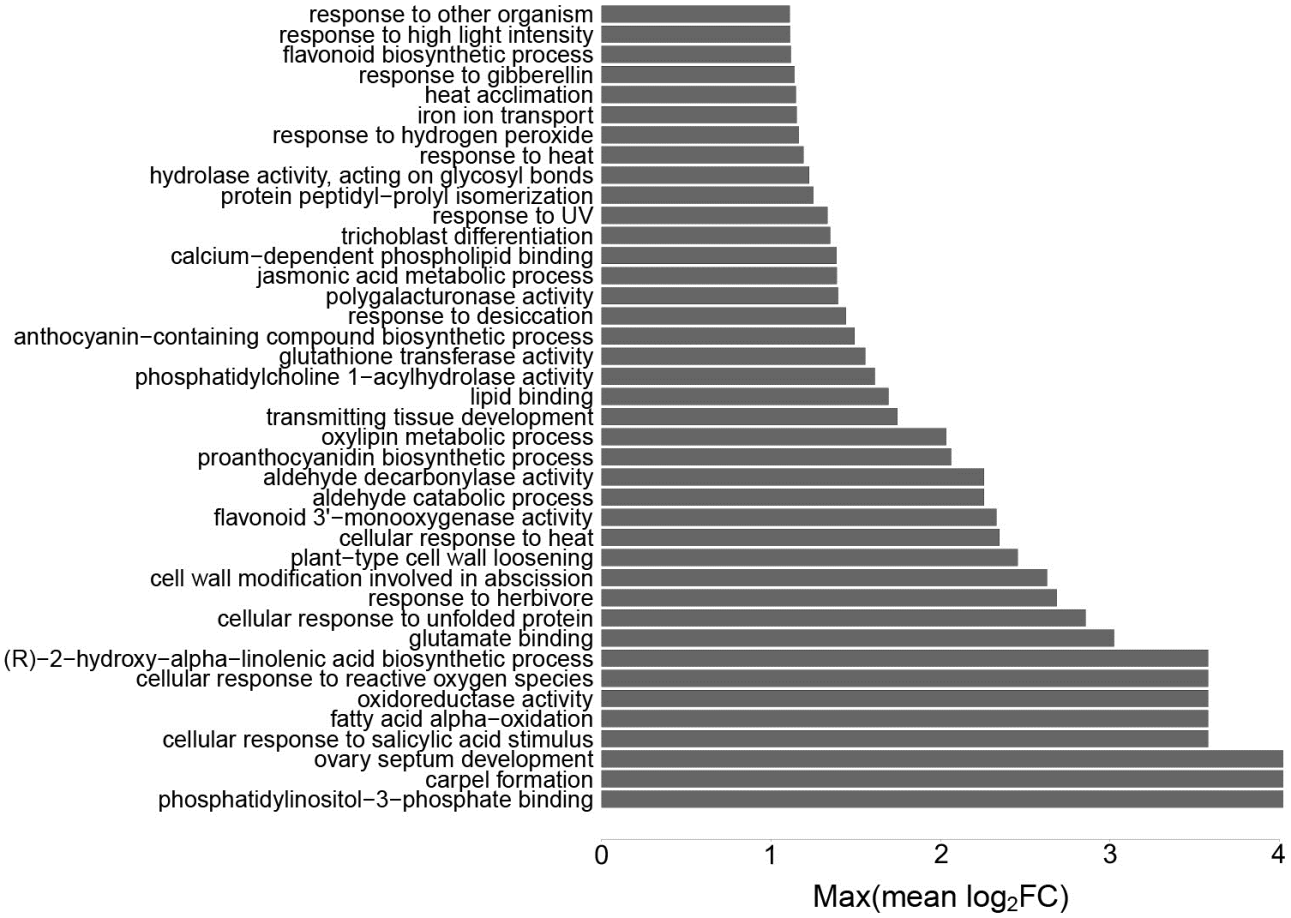
Top 40 pathways with highest differential expression levels in heat stress. Gene set analysis (GSA) identified a set of significantly enriched GOs, characterized in terms of maxmean statistic. Y-axis shows the significantly enriched GO terms, and x-axis is the maxmean statistic of the gene set expressed in terms of log_2_FC (S2 Table).

In heat stress, 88 out of 136 significantly enriched GO categories were expressed at higher levels than in control (Fig 6 and S2 Table). A majority of these categories were related to plant immune responses or fatty acid biogenesis (S2 Fig).

Plant cell wall synthesis is an important factor in plants for coping with different stresses. The dynamic nature of cell wall synthesis ensures the plant resistance to stresses, and supports the growth and development of the plant [41–43]. Four processes related to cell wall synthesis were enriched in all three conditions (H, D and HD), with mean expression increase by 2.1 fold (S2 Table). For more detailed analysis, we inspected 48 genes with a putative ortholog reported to take part in cell wall biogenesis [43] (S9 Table). Expression patterns of these genes show the active nature of cell wall biogenesis in response to heat, drought and combined heat and drought conditions.

The abundance of glucuronic acid increased significantly in all conditions, by three fold in heat and drought conditions and 8.6 fold in combined heat and drought. Glucuronic acid is utilized by different pathways such as biosynthesis of arabinose, xylose, galacturonic acid, and apiose residues found in cell wall constituents such as pectin and hemicellulose [44], suggesting that cell wall-related processes were activated (S7 Table). In transcriptomic data, “polygalacturonase activity” (pectin biosynthesis) was enriched in *P*. *dactylifera* in heat stress with mean fold change of 2.6. Altogether, these data sets together suggest active remodeling of the cell wall.

#### Combined heat and drought

**Fig 7:**
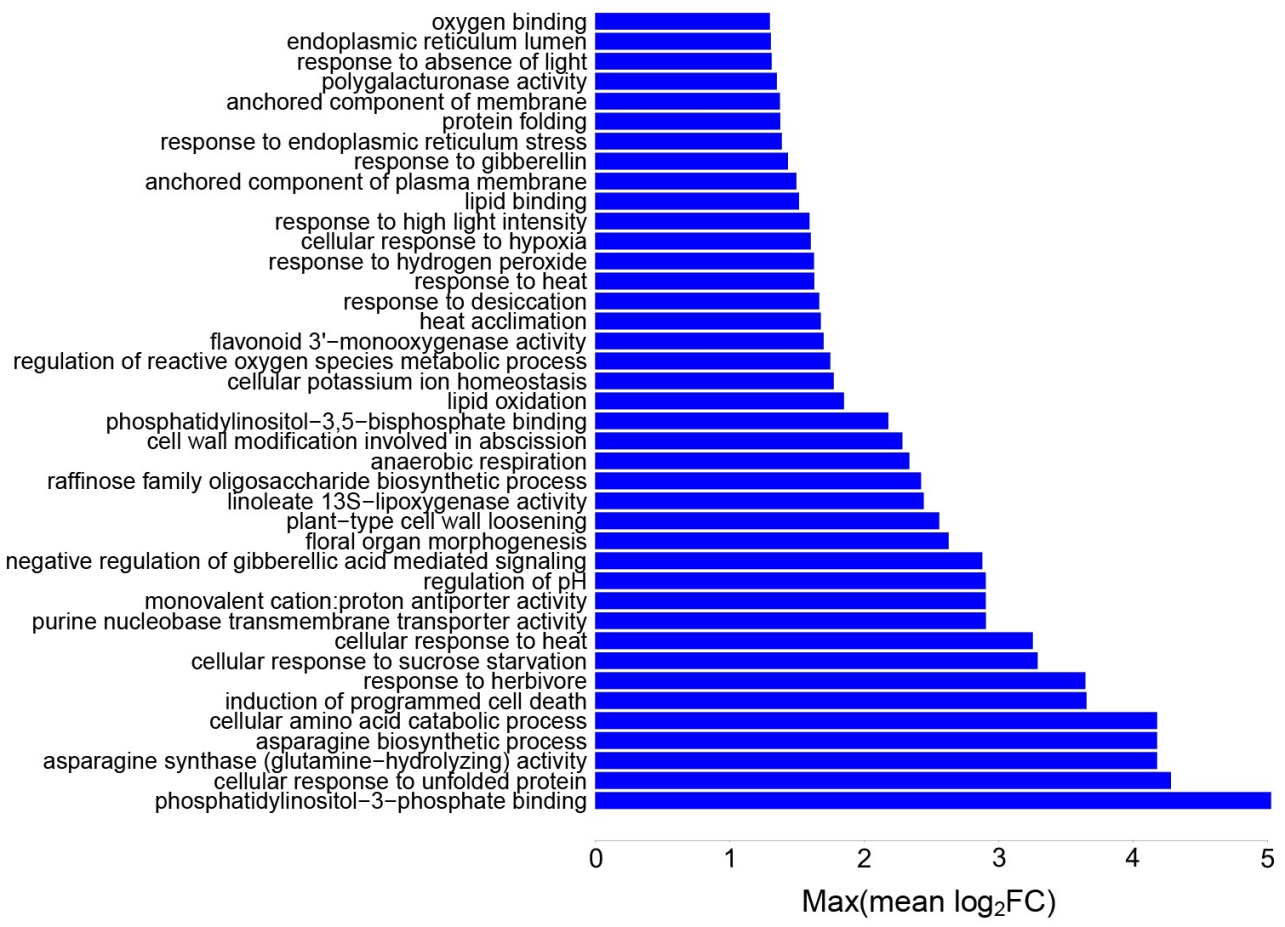
Top 40 pathways with highest expression levels in combined heat and drought stress. Gene set analysis (GSA) identified a set of significantly enriched GOs, characterized in terms of maxmean statistic. Y-axis shows the significantly enriched GO terms, and x-axis is the maxmean statistic of the gene set expressed in terms of log_2_FC (S2 Table).

In the combined heat and drought stress, 195 significantly enriched pathways were identified from transcriptomic data. Out of these, 57 GO categories were positively enriched with mean log_2_FC>0 (Fig 7). The associated pathways were protein folding and unfolding, plant immune responses, heat acclimation, response to phytohormones, hydrogen peroxide, cell wall biogenesis, programmed cell death and secondary metabolism (S3 Fig). Altogether 99 GO categories were unique to combined heat and drought (S2 Table).

The expression of “wax biosynthetic process” was increased by two fold in combined heat and drought. *P*. *dactylifera* has 30 genes annotated to be involved in wax biosynthesis (S2 Table). Intra or epicuticular wax production is an effective physical barrier against abiotic and even biotic stresses. In case of abiotic stresses, wax is known to have beneficial effects by preventing water loss (drought stress) and reflecting the excess radiation (heat stress). This protective shield creates an isolation coat over the leaf surface in harsh arid climates [45].

In metabolomics data, galactose content was increased eight fold in combined heat and drought. Soluble carbohydrates such as galactose are known to accumulate during drought stress [46]. UDP glucose 4-epimerase is an important enzyme of galactose metabolic pathway which facilitates the reversible conversion between UDP galactose and UDP glucose. In *A*. *thaliana*, overexpression of rice UDP glucose 4-epimerase in a transgenic line resulted in tolerance to salt, drought, and freezing stress [47]. UDP glucose 4-epimerase regulates the monosaccharide pool available for pectin production [48], but also plays an important role in galactinol, stachyose, and raffinose biosynthesis. There are 14 paralogous UDP glucose 4-epimerase genes in *P*. *dactylifera*, one of which showed significantly differential expression levels in both heat and combined heat and drought (PDACT_KE332782.1_G000390), and two paralogs which were differentially expressed only in combined heat and drought (PDACT_KE332624.1_G000480 and PDACT_KE332831.1_G001110).

### Motif enrichment

**Fig 8:**
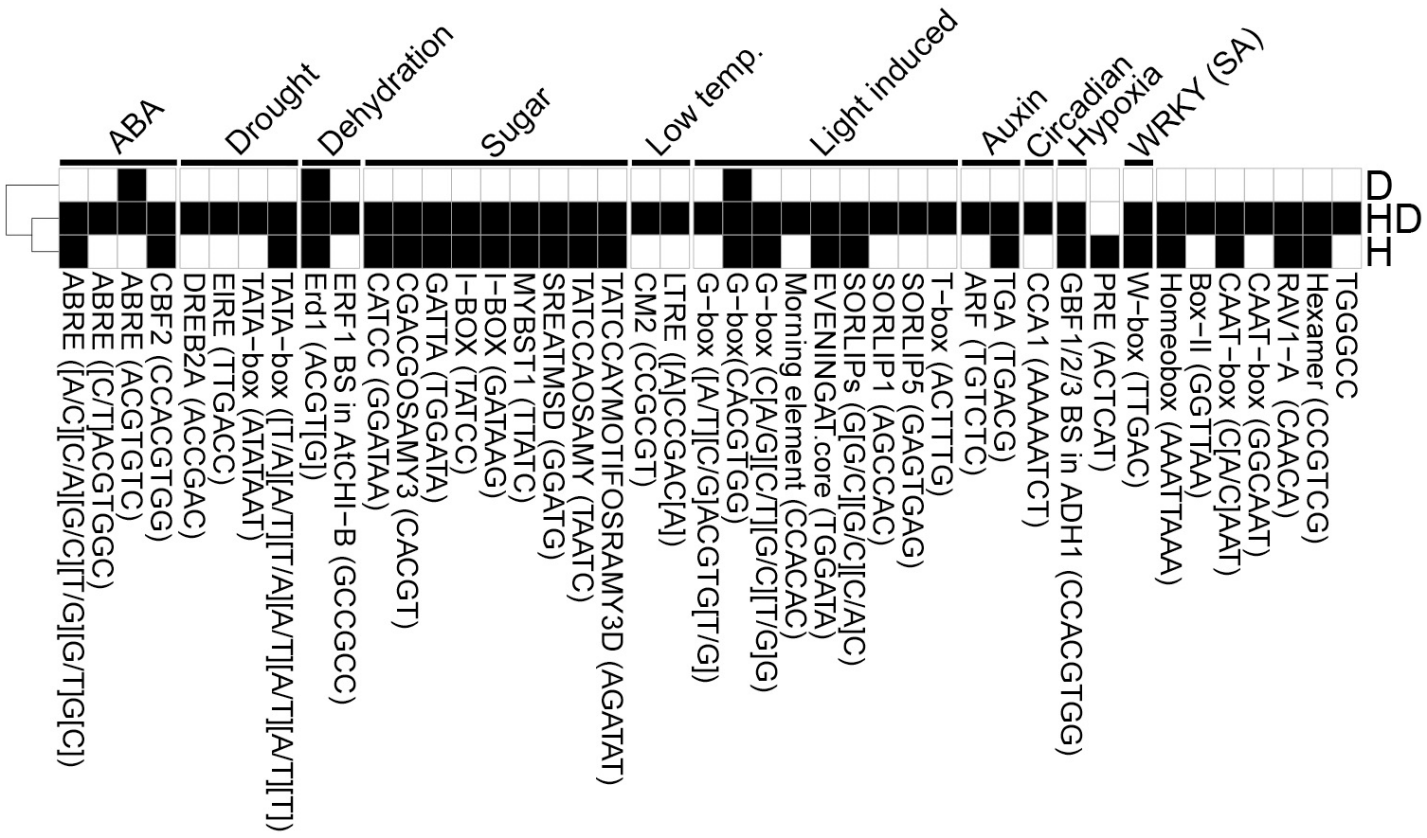
Motif enrichment in the differentially expressed genes. Known *cis*-motifs were identified in the promoter regions 1000 bp upstream of the genes, and analyzed for enrichment using Fisher test. The p-values were corrected for multiple testing by Benjamini-Hochberg FDR correction. Black color: FDR ≤ 0.05 (significant enrichment), white: non-significant. Columns are motifs and their sequences, annotated to an associated biological process. Rows are heat, drought and combined heat and drought (H, D, HD) experiments (S3 Table).

Motif enrichment analysis revealed a higher amount of enriched motifs in combined heat and drought and heat conditions, and to a lesser degree in drought (Fig 8, S3 Table). Interestingly, all *cis*-acting elements involved in sugar regulation [33] were significantly enriched in the heat and combined heat and drought conditions, suggesting that sugar signaling has an eminent role in the date palm gene regulation related to heat stress.

A significant enrichment of circadian motif and morning element-containing genes was detected in heat and combined heat and drought. Association between the stress-induced genes and circadian rhythm and diurnal cycle could be evidence of a stress avoidance strategy in date palm; stress reactions could be triggered in the morning to improve the palm tolerance to the harsh conditions during daytime. Further evidence of this is that from the 57 HSP genes with significantly increased expression in the experimental conditions (S6 Table), 27 genes contained the circadian clock associated 1 (CCA1, seq: AAAAATCT) promoter motif (FDR ≤ 0.05). In addition, two out of these 27 HSPs contained also a morning element (CCACAC), 10 genes had an evening element (TGGATA), and one gene had both morning and evening elements (S6 Table). The CCA1-motif containing genes are involved in adjustment to environmental conditions, such as light and temperature, and they regulate specific biological processes synchronized to a particular time of the day. We found no CCA1 nor evening element motifs in *A*. *thaliana* HSP promoters, but 14 HSP orthologues contained morning element motifs. A complete set of enriched GOs for the genes containing circadian and light responsive *cis*-motifs is in S4 Table.

Proline is involved in several stress responses as an osmolyte [49], a signaling molecule [50], a metal chelator [51] through chaperone activities, and in direct ROS scavenging [52]. Proline was significantly increased by 9.5 fold in the drought experiment; in combined heat and drought the increase was 4.3, but marginally below the level of statistical significance (FDR = 0.0576, S7 Table). The PRE motif (ACTCAT, Proline and hypoosmolarity responsive) was enriched among differentially expressed genes in the heat experiment (Fig 8, S3 Table), and in transcriptomics, GSA analysis showed enrichment of “proline transport” with a two fold increase in the heat experiment (S2 Table). Interestingly, the proline levels were not altered in the heat stress, which could suggest that proline act as a signaling molecule in heat stress.

To dissect the processes regulated by diurnal cycle in the different stress conditions, further enrichment analysis of these genes was carried out. Overall, GO enrichment analysis of all genes containing circadian and light responsive, sugar, drought and ABA *cis*-regulatory elements showed seven common enriched GOs: vacuole, plasma membrane, response to heat, water deprivation, high light, endoplasmic reticulum stress and hydrogen peroxide. Out of these, 5 GOs (response to heat, water deprivation, high light, endoplasmic reticulum stress and hydrogen peroxide) were significantly enriched in the set of differentially expressed genes in the D, H, or HD experiments.

Furthermore, ABA regulatory elements were enriched in both heat and combined heat and drought, but not in drought. ABA is generally induced under osmotic stresses and is known to be a positive regulator of LEA class proteins, heat shock proteins and protein phosphatase 2C (PP2Cs) [53]. No enrichment in drought could result from the fact that the conditions were not severe enough for the date palm to elicit a drought response. We further detected the WRKY W-box element to be significantly enriched in the combined heat and drought and heat. WRKY transcription factor family is a key regulator in many processes such as response to biotic and abiotic stresses, and senescence [54]. The drought responsive element was not enriched in any condition.

## Conclusion

We have studied the stress adaptation strategies initiated in date palm, *Phoenix dactylifera*, under mild heat, drought, and combined heat and drought conditions by transcriptomic and metabolomic profiling. Overall the transcriptional profile showed that combined heat and drought and heat had more similar response, whereas drought resembled control. Other monocot species have shown a similar balance tipped towards heat (*T*. *aestivum* and *S*. *bicolor)* [2]. In the metabolomics data the balance was opposite, with the combined heat and drought resembling the drought condition. This could be due to a more immediate response to water deficiency at the metabolomic level, which leads the plant to switch more towards carbohydrate metabolism. Based on the transcriptomic data and the overall levels of hydrogen peroxide, the date palm did not appear to be very stressed, since ROS was effectively compartmentalized and scavenged. Therefore it could be that many of the observed responses at the transcriptional level do not necessarily lead into translation of the proteins.

Overall, it appears that date palm reacts to drought and heat conditions in a similar manner to other plants. The hallmarks of heat stress were visible in the transcriptomics data, such as protein misfolding, response to hydrogen peroxide and cell wall modification, as well as ABA signaling in the case of drought. Since the plants were exposed to the stress for several days before harvesting, the early signs of heat stress such as calcium and NO signaling were not detected anymore. Interestingly, two orthologs of heat shock factor HSFA2 showed increased expression in all three conditions. They are known to be the most highly heat-induced heat shock factor in Arabidopsis, and also to play an important role in recovery from heat shock [55] and extension of thermotolerance in Arabidopsis [56].

Finally, we detected a significant enrichment of circadian rhythm motifs in the differentially expressed genes in heat and combined heat and drought stresses, suggesting new stress avoidance strategies. For example, some heat shock factors contain motifs for circadian regulation in the promoter regions, making it possible to synchronize HSP production to the time of the day when they are needed. A similar connection has been reported in Arabidopsis in the case of negative regulators of freezing tolerance, COR27 and COR28 [54].

## Supporting information

S1 Table Samples and their sequencing statistics. (XLSX)

S2 Table Significantly enriched GOs in different treatments. (XLSX)

S3 Table Enrichment analysis of the regulatory motifs among the differentially expressed genes in drought, heat, and combined heat and drought treatments. (XLSX)

S4 Table GO enrichment analysis of genes containing CCA1, light responsive, ABA, drought and sugar motifs in their promoter regions. (XLSX)

S5 Table Expressed genes (24504 genes) and their differential expression values. (XLSX)

S6 Table Differentially expressed heat shock protein genes in drought, heat, and combined heat and drought experiments. (XLSX)

S7 Table Differentially abundant metabolites in the heat, drought, and combined heat and drought treatments, and their annotations. (XLSX)

S8 Table Differentially expressed genes encoding ROS and redox-related enzymes in heat, drought, or combined heat and drought treatments. (XLSX)

S9 Table Genes putatively associated with cell wall biogenesis and their differential expression. (XLSX)

S10 Table Putative orthologs genes from *A*. *thaliana* mapped to *P*. *dactylifera*, and tested for enrichment among the differentially expressed genes (abs(log2FC) ≥ 1) using Fisher exact test. (XLSX)

S1 Fig Treemap of significantly enriched (Log_2_FC > 0) Gene Ontology terms in drought. (PDF)

S2 Fig Treemap of significantly enriched (Log_2_FC > 0) Gene Ontology terms in heat. (PDF)

S3 Fig Treemap of significantly enriched (Log_2_FC > 0) Gene Ontology terms in combined heat and drought. (PDF)

## References

[1] Rasmussen S, Barah P, Suarez-Rodriguez MC, Bressendorff S, Friis P, Costantino P, et al Transcriptome responses to combinations of stresses in Arabidopsis. Plant Physiol. 2013; 161(4): 1783–94. 10.1104/pp.112.210773 23447525 PMC3613455

[2] Pandey P, Ramegowda V, Senthil-Kumar M. Shared and unique responses of plants to multiple individual stresses and stress combinations: physiological and molecular mechanisms. Front Plant Sci. 2015; 6: 723–37. 10.3389/fpls.2015.00723 26442037 PMC4584981

[3] Nankishore A, Farrell AD. The response of contrasting tomato genotypes to combined heat and drought stress. J Plant Physiol. 2016; 202: 75–82. 10.1016/j.jplph.2016.07.006 27467552

[4] Saidi Y, Finka A, Goloubinoff P. Heat perception and signalling in plants: a tortuous path to thermotolerance. New Phytol. 2011; 190(3): 556–65. 10.1111/j.1469-8137.2010.03571.x 21138439

[5] Jacob P, Hirt H, Bendahmane A. The heat-shock protein/chaperone network and multiple stress resistance. Plant Biotechnol J. 2017; 15(4): 405–14. 10.1111/pbi.12659 27860233 PMC5362687

[6] Penfield S. Temperature perception and signal transduction in plants. New Phytol. 2008; 179(3): 615–28. 10.1111/j.1469-8137.2008.02478.x 18466219

[7] Feller U. Drought stress and carbon assimilation in a warming climate: Reversible and irreversible impacts. J Plant Physiol. 2016; 203: 84–94. 10.1016/j.jplph.2016.04.002 27083537

[8] Osakabe Y, Osakabe K, Shinozaki K, Tran LS. Response of plants to water stress. Front Plant Sci. 2014; 5: 86–94. 10.3389/fpls.2014.00086 24659993 PMC3952189

[9] Zohary D, Hopf M, Weiss E. Domestication of Plants in the Old World: The origin and spread of domesticated plants in south-west Asia, Europe, and the Mediterranean Basin, Fourth Edition 2012.

[10] Arab L, Kreuzwieser J, Kruse J, Zimmer I, Ache P, Alfarraj S, et al Acclimation to heat and drought—Lessons to learn from the date palm (*Phoenix dactylifera*). Env Exp Bot. 2016; 125: 20–30.

[11] Kruse J, Adams MA, Kadinov G, Arab L, Kreuzwieser J, Alfarraj S, et al Characterization of photosynthetic acclimation in *Phoenix dactylifera* by a modified Arrhenius equation originally developed for leaf respiration. Trees. 2017; 31(2): 623–44.

[12] Lytovchenko A, Beleggia R, Schauer N, Isaacson T, Leuendorf JE, Hellmann H, et al Application of GC-MS for the detection of lipophilic compounds in diverse plant tissues. Plant methods. 2009; 5: 4–15. 10.1186/1746-4811-5-4 19393072 PMC2680844

[13] Jaeger C, Gessler A, Biller S, Rennenberg H, Kreuzwieser J. Differences in C metabolism of ash species and provenances as a consequence of root oxygen deprivation by waterlogging. J Exp Bot. 2009; 60(15): 4335–45. 10.1093/jxb/erp268 19717531

[14] Hummel J, Strehmel N, Selbig J, Walther D, Kopka J. Decision tree supported substructure prediction of metabolites from GC-MS profiles. Metabolomics. 2010; 6(2): 322–33. 10.1007/s11306-010-0198-7 20526350 PMC2874469

[15] Styczynski MP, Moxley JF, Tong LV, Walther JL, Jensen KL, Stephanopoulos GN. Systematic identification of conserved metabolites in GC/MS data for metabolomics and biomarker discovery. Anal Chem. 2007; 79(3): 966–73. 10.1021/ac0614846 17263323

[16] Robinson MD, McCarthy DJ, Smyth GK. edgeR: a Bioconductor package for differential expression analysis of digital gene expression data. Bioinformatics. 2010; 26(1): 139–40. 10.1093/bioinformatics/btp616 19910308 PMC2796818

[17] Bemm F, Becker D, Larisch C, Kreuzer I, Escalante-Perez M, Schulze WX, et al Venus flytrap carnivorous lifestyle builds on herbivore defense strategies. Genome Res. 2016; 26(6): 812–25. 10.1101/gr.202200.115 27197216 PMC4889972

[18] Bolger AM, Lohse M, Usadel B. Trimmomatic: a flexible trimmer for Illumina sequence data. Bioinformatics. 2014; 30(15): 2114–20. 10.1093/bioinformatics/btu170 24695404 PMC4103590

[19] Al-Mssallem IS, Hu S, Zhang X, Lin Q, Liu W, Tan J, et al Genome sequence of the date palm *Phoenix dactylifera* L. Nature communications. Nat Commun. 2013; 4: 2274–83. 10.1038/ncomms3274 23917264 PMC3741641

[20] Bray NL, Pimentel H, Melsted P, Pachter L. Near-optimal probabilistic RNA-seq quantification. Nat Biotechnol. 2016; 34(5): 525–7. 10.1038/nbt.3519 27043002

[21] Goodstein DM, Shu S, Howson R, Neupane R, Hayes RD, Fazo J, et al Phytozome: a comparative platform for green plant genomics. Nucleic Acids Res. 2012; 40: 1178–86. 10.1093/nar/gkr944 PMC3245001 22110026

[22] Emms DM, Kelly S. OrthoFinder: solving fundamental biases in whole genome comparisons dramatically improves orthogroup inference accuracy. Genome Biol. 2015; 16: 157–71. 10.1186/s13059-015-0721-2 26243257 PMC4531804

[23] Willems P, Mhamdi A, Stael S, Storme V, Kerchev P, Noctor G, et al The ROS Wheel: refining ROS transcriptional footprints. Plant Physiol. 2016; 171(3): 1720–33. 10.1104/pp.16.00420 27246095 PMC4936575

[24] Mittler R, Vanderauwera S, Gollery M, Van Breusegem F. Reactive oxygen gene network of plants. Trends Plan Sci. 2004; 9(10): 490–8. 10.1016/j.tplants.2004.08.009 15465684

[25] Camacho C, Coulouris G, Avagyan V, Ma N, Papadopoulos J, Bealer K, et al BLAST plus: architecture and applications. BMC Bioinformatics. 2009; 10: 421–30. 10.1186/1471-2105-10-421 20003500 PMC2803857

[26] Tang H, Klopfenstein D, Pedersen B, Flick P, Sato K, Ramirez F, et al. GOATOOLS: Tools for Gene Ontology. Zenodo. 2015.

[27] Varemo L, Nielsen J, Nookaew I. Enriching the gene set analysis of genome-wide data by incorporating directionality of gene expression and combining statistical hypotheses and methods. Nucleic Acids Res. 2013; 41(8): 4378–91. 10.1093/nar/gkt111 23444143 PMC3632109

[28] Supek F, Bosnjak M, Skunca N, Smuc T. REVIGO Summarizes and Visualizes Long Lists of Gene Ontology Terms. PlosOne. 2011; 6(7): e21800. 10.1371/journal.pone.0021800 PMC3138752 21789182

[29] Davuluri RV, Sun H, Palaniswamy SK, Matthews N, Molina C, Kurtz M, et al AGRIS: Arabidopsis Gene Regulatory Information Server, an information resource of Arabidopsis *cis*-regulatory elements and transcription factors. BMC Bioinformatics. 2003; 4(1): 25. 12820902 10.1186/1471-2105-4-25 PMC166152

[30] Higo K, Ugawa Y, Iwamoto M, Korenaga T. Plant *cis*-acting regulatory DNA elements (PLACE) database: 1999. Nucleic Acids Res. 1999; 27(1): 297–300. 9847208 10.1093/nar/27.1.297 PMC148163

[31] Ming R, VanBuren R, Wai CM, Tang H, Schatz MC, Bowers JE, et al The pineapple genome and the evolution of CAM photosynthesis. Nat Genet. 2015; 47(12): 1435–42. 10.1038/ng.3435 26523774 PMC4867222

[32] Michael S, Trave G, Ramu C, Chica C, Gibson TJ. Discovery of candidate KEN-box motifs using cell cycle keyword enrichment combined with native disorder prediction and motif conservation. Bioinformatics. 2008; 24(4): 453–7. 10.1093/bioinformatics/btm624 18184688

[33] Cordoba E, Aceves-Zamudio DL, Hernandez-Bernal AF, Ramos-Vega M, Leon P. Sugar regulation of SUGAR TRANSPORTER PROTEIN 1 (STP1) expression in *Arabidopsis thaliana*. J Exp Bot. 2015; 66(1): 147–59. 10.1093/jxb/eru394 25281700 PMC4265152

[34] Nishizawa A, Yabuta Y, Yoshida E, Maruta T, Yoshimura K, Shigeoka S. Arabidopsis heat shock transcription factor A2 as a key regulator in response to several types of environmental stress. Plant J. 2006; 48(4): 535–47. 10.1111/j.1365-313X.2006.02889.x 17059409

[35] Watanabe N, Lam E. BAX inhibitor-1 modulates endoplasmic reticulum stress-mediated programmed cell death in Arabidopsis. J Biol Chem. 2008; 283(6): 3200–10. 10.1074/jbc.M706659200 18039663

[36] Tremblay A, Seabolt S, Zeng H, Zhang C, Bockler S, Tate DN, et al A role of the Fuzzy Onions Like gene in regulating cell death and defense in Arabidopsis. Sci Rep. 2016; 6: 37797–809. 10.1038/srep37797 27898102 PMC5127180

[37] Echevarria-Zomeno S, Fernandez-Calvino L, Castro-Sanz AB, Lopez JA, Vazquez J, Castellano MM. Dissecting the proteome dynamics of the early heat stress response leading to plant survival or death in Arabidopsis. Plant Cell Environ. 2016; 39(6): 1264–78. 10.1111/pce.12664 26580143

[38] Wang X, Cai X, Xu C, Wang Q, Dai S. Drought-responsive mechanisms in plant leaves revealed by proteomics. Int J Mol Sci. 2016; 17(10): 1706–36. 10.3390/ijms17101706 PMC5085738 27763546

[39] Park C-J, Seo Y-S. Heat Shock Proteins: A review of the molecular chaperones for plant immunity. Plant Pathol J. 2015; 31(4): 323–33. 10.5423/PPJ.RW.08.2015.0150 26676169 PMC4677741

[40] Martinez IM, Chrispeels MJ. Genomic analysis of the unfolded protein response in Arabidopsis shows its connection to important cellular processes. Plant Cell. 2003; 15(2): 561–76. 10.1105/tpc.007609 12566592 PMC141221

[41] Cosgrove DJ. Catalysts of plant cell wall loosening. F1000Research. 2016; 5: 119–37. 10.12688/f1000research.7180.1 PMC4755413 26918182

[42] Huot B, Yao J, Montgomery BL, He SY. Growth-defense tradeoffs in plants: a balancing act to optimize fitness. Mol Plant. 2014; 7(8): 1267–87. 10.1093/mp/ssu049 24777989 PMC4168297

[43] Le Gall H, Philippe F, Domon JM, Gillet F, Pelloux J, Rayon C. Cell wall metabolism in response to abiotic stress. Plants. 2015; 4(1): 112–66. 10.3390/plants4010112 27135320 PMC4844334

[44] Reboul R, Geserick C, Pabst M, Frey B, Wittmann D, Lutz-Meindl U, et al Down-regulation of UDP-glucuronic acid biosynthesis leads to swollen plant cell walls and severe developmental defects associated with changes in pectic polysaccharides. J Biol Chem. 2011; 286(46): 39982–92. 10.1074/jbc.M111.255695 21949134 PMC3220558

[45] Khelil R, Jardé E, Cabello-Hurtado F, Ould-el-Hadj Khelil A, Esnault M-A. Structure and composition of the wax of the date palm, *Phoenix dactylifera* L., from the septentrional Sahara. Scientia Hortic. 2016; 201: 238–46.

[46] Evers D, Lefevre I, Legay S, Lamoureux D, Hausman JF, Rosales ROG, et al Identification of drought-responsive compounds in potato through a combined transcriptomic and targeted metabolite approach. J Exp Bot. 2010; 61(9): 2327–43. 10.1093/jxb/erq060 20406784

[47] Liu H-l, Dai X-y, Xu Y-y, Chong K. Over-expression of OsUGE-1 altered raffinose level and tolerance to abiotic stress but not morphology in Arabidopsis. J Plant Physiol. 2007; 164(10): 1384–90. 10.1016/j.jplph.2007.03.005 17566602

[48] Reiter WD, Vanzin GF. Molecular genetics of nucleotide sugar interconversion pathways in plants. Plant Mol Biol. 2001; 47(1–2): 95–113. 11554483

[49] Yoshiba Y, Kiyosue T, Nakashima K, YamaguchiShinozaki K, Shinozaki K. Regulation of levels of proline as an osmolyte in plants under water stress. Plant Cell Physiol. 1997; 38(10): 1095–102. 9399433 10.1093/oxfordjournals.pcp.a029093

[50] Szabados L, Savoure A. Proline: a multifunctional amino acid. Trends Plan Sci. 2010; 15(2): 89–97. 10.1016/j.tplants.2009.11.009 20036181

[51] Farago ME, Mullen WA. Plants which accumulate metals. Part IV. A possible copper-proline complex from the roots of *armeria maritima*. Inorg Chim Acta. 1979; 32: L93–L94.

[52] Liang X, Zhang L, Natarajan SK, Becker DF. Proline mechanisms of stress survival. Antioxid Redox Signal. 2013; 19(9): 998–1011. 10.1089/ars.2012.5074 23581681 PMC3763223

[53] Nakashima K, Yamaguchi-Shinozaki K. ABA signaling in stress-response and seed development. Plant Cell Rep. 2013; 32(7): 959–70. 10.1007/s00299-013-1418-1 23535869

[54] Li X, Ma D, Lu SX, Hu X, Huang R, Liang T, et al Blue light- and low temperature-regulated COR27 and COR28 play roles in the Arabidopsis circadian clock. Plant Cell. 2016; 28(11): 2755–69. 10.1105/tpc.16.00354 27837007 PMC5155342

[55] Jacob P, Hirt H, Bendahmane A. The heat-shock protein/chaperone network and multiple stress resistance. Plant Biotechnol J. 2017; 15(4): 405–14. 10.1111/pbi.12659 27860233 PMC5362687

[56] Charng Y-y, Liu H-c, Liu N-y, Chi W-t, Wang C-n, Chang S-h, et al A heat-inducible transcription factor, HsfA2, is required for extension of acquired thermotolerance in Arabidopsis. Plant Physiol. 2007; 143(1): 251–62. 10.1104/pp.106.091322 17085506 PMC1761974

