# Supplementary figures and images for: Detecting early signs of heat and drought stress in Phoenix dactylifera (date palm)
Source: PLoS One. 2017 Jun 1;12(6):e0177883. doi: 10.1371/journal.pone.0177883 (PMC5453443; doi:10.1371/journal.pone.0177883)

Treemap of significantly upregulated Gene Ontology terms in drought

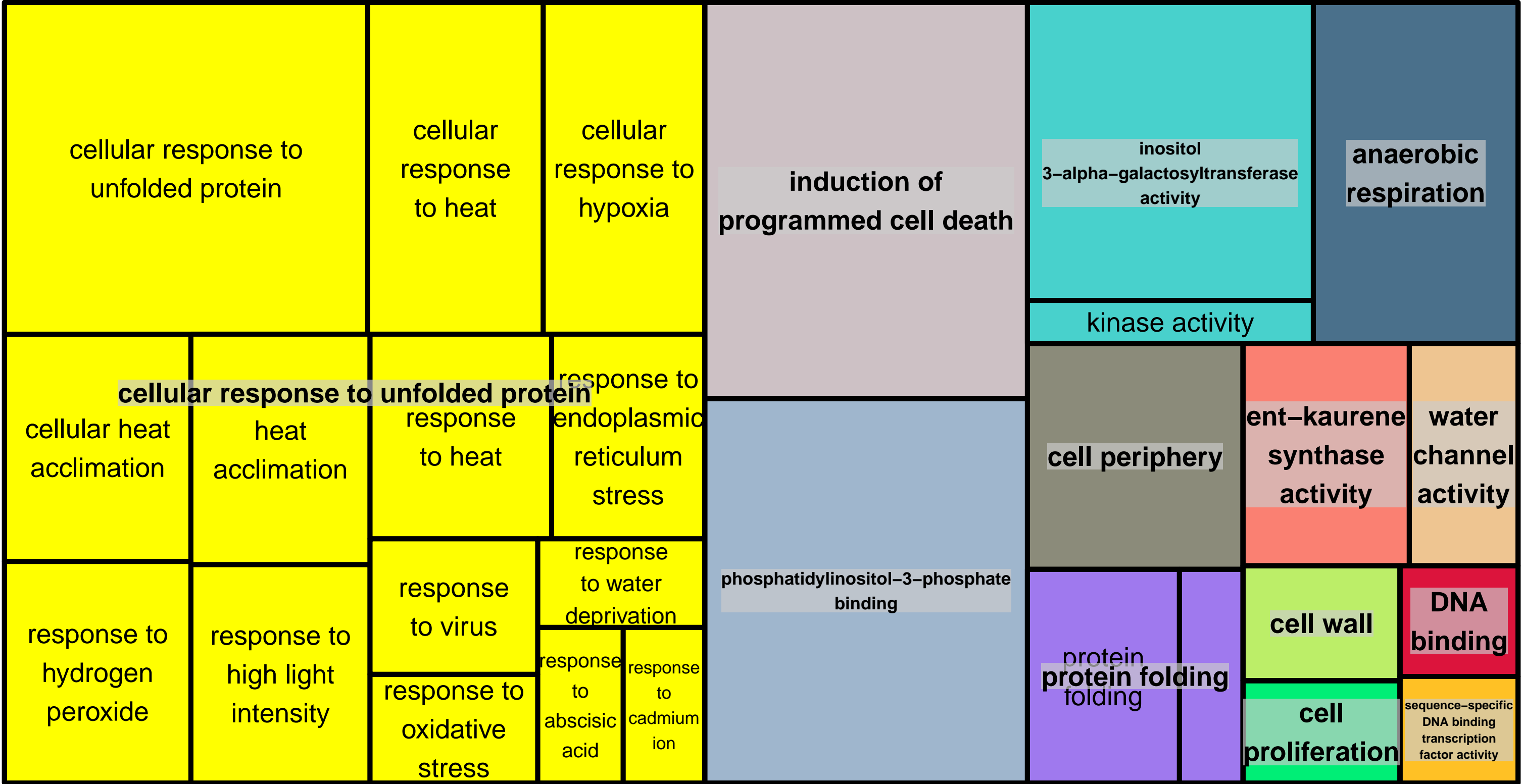

Supplement: S1 Fig — (PDF) [file pone.0177883.s011.pdf]

# Treemap of significantly upregulated Gene Ontology terms in heat

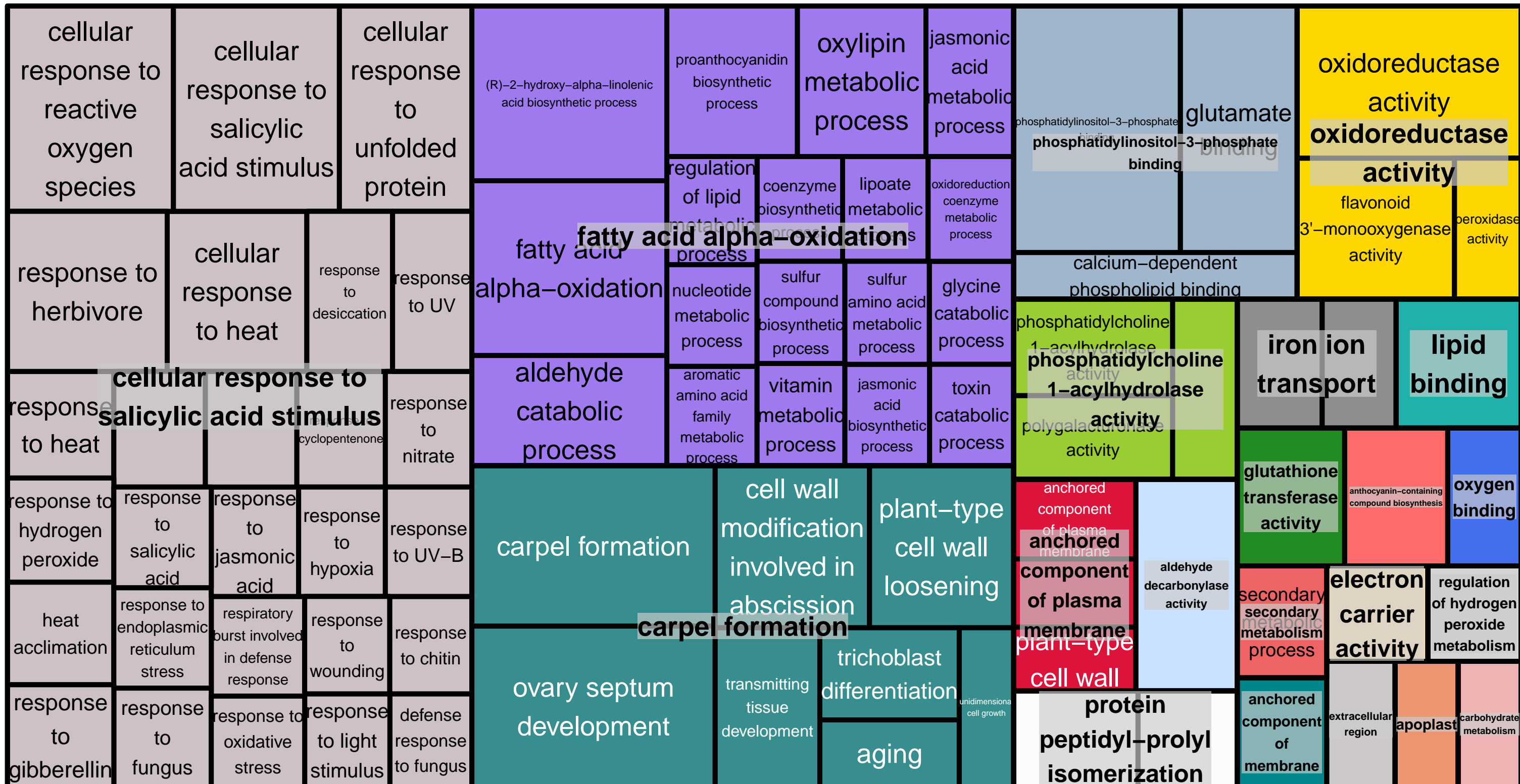

Supplement: S2 Fig — (PDF) [file pone.0177883.s012.pdf]

Treemap of significantly upregulated Gene Ontology terms in combined heat and drought

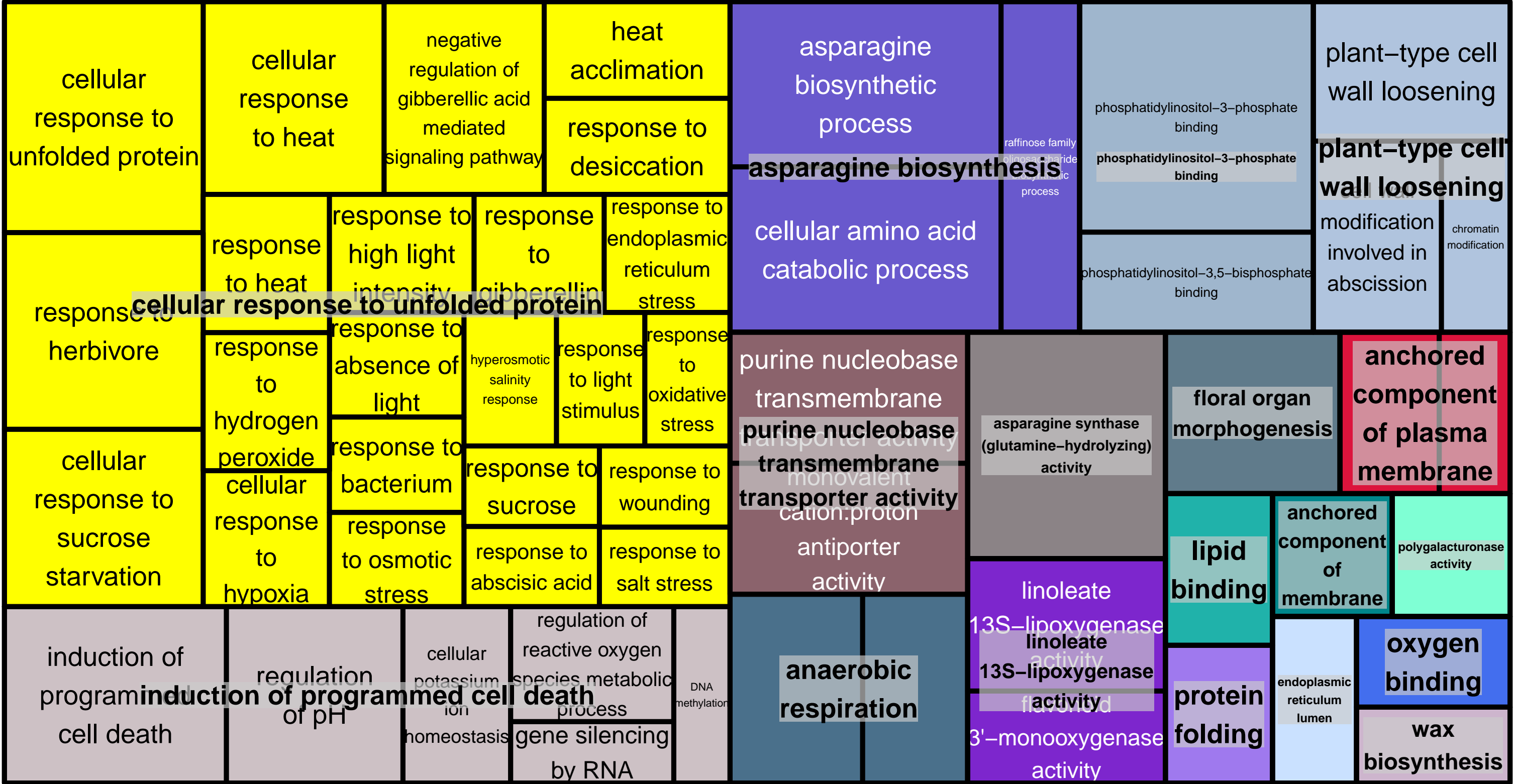

Supplement: S3 Fig — (PDF) [file pone.0177883.s013.pdf]
